# Low-level neurostimulation of the renal nerves as a potential therapeutic strategy for hypertension treatment

**DOI:** 10.3389/fphar.2025.1529844

**Published:** 2025-02-26

**Authors:** Ibrahim M. Salman, Omar Z. Ameer, Sarah F. Hassan, Arun Sridhar, Yee-Hsee Hsieh, Stephen J. Lewis

**Affiliations:** ^1^ Department of Pharmaceutical Sciences, College of Pharmacy, Alfaisal University, Riyadh, Saudi Arabia; ^2^ Department of Disease Biology, Galvani Bioelectronics, Stevenage, Hertfordshire, United Kingdom; ^3^ Division of Pulmonary, Critical Care, and Sleep Medicine, School of Medicine, Case Western Reserve University, Cleveland, OH, United States; ^4^ Division of Pulmonology, Allergy and Immunology, Department of Pediatrics, School of Medicine, Case Western Reserve University, Cleveland, OH, United States; ^5^ Electrical Stimulation Center, Case Western Reserve University, Cleveland, OH, United States

**Keywords:** neurostimulation, renal nerve stimulation, renal innervation, hypertension, spontaneously hypertensive rat

## Abstract

**Background:**

Neurostimulation is an emerging treatment for conditions like hypertension. The renal nerves, comprising sensory afferent and sympathetic efferent fibers, are crucial for blood pressure (BP) regulation. The inhibitory reno-renal reflex, where central integration of renal sensory input reduces sympathetic outflow and systemic BP, presents a promising target for neurostimulation interventions. We therefore investigated renal nerve stimulation (RNS) as a potential hypertension therapy.

**Methods:**

Anesthetized male spontaneously hypertensive rats (SHRs) were subjected to low-level RNS at 0.5 mA pulse amplitude and 0.5 ms pulse width for 30 s delivered to the left intact renal nerve at 2.5 and 5.0 Hz. Mean arterial pressure (MAP), heart rate (HR), hindquarter blood flow (HQF), and ipsilateral renal cortical blood flow (RCF) were recorded. Hindquarter resistance (HQR) and renal cortical resistance (RCR) were derived from MAP and flow values.

**Results:**

RNS significantly reduced MAP, with similar depressor responses at 2.5 (27 ± 3 mmHg) and 5.0 Hz (37 ± 8 mmHg). RNS substantially increased HQF and reduced HQR, with comparable effects at both frequencies. A 5-Hz stimulus markedly reduced RCF and increased RCR of the ipsilateral kidney. When the stimulation frequency was lowered to 2.5 Hz, the changes in RCF and RCR were nearly indistinguishable from baseline.

**Conclusion:**

Low-level RNS effectively lowers BP in the SHR model of hypertension and may offer a promising therapeutic alternative for hypertension treatment. Physiologically, the observed clinically relevant reductions in BP were primarily due to reductions in vascular resistance. Adjusting stimulus levels can achieve desired hypotensive responses without compromising ipsilateral renal blood supply, typically affected by direct renal sympathetic fiber stimulation.

## Introduction

Hypertension affects more than 1 billion people worldwide and is a predisposing factor to many cardiovascular complications such as stroke, cardiac hypertrophy, myocardial infarction, heart failure and renal failure (Carey et al., 2022; Dzau and Hodgkinson, 2024). Despite the development of various antihypertensive drugs, the number of patients living with uncontrolled hypertension continues to rise (Shalaeva and Messerli, 2023). Identifying novel approaches for the treatment of high blood pressure (BP) remains crucial for reducing the global burden of cardiovascular disease and improving patient outcomes.

Renal innervation, including “efferent” sympathetic and “afferent” sensory neurons, plays a central role in the regulation of body fluid homeostasis and arterial BP. Renal sympathetic nerves innervate major structural components such as blood vessels, tubules, the renal pelvis, and glomeruli, and their activity plays an important role in regulating glomerular filtration rate (GFR), sodium and water reabsorption, renin release, vascular resistance (VR), and ultimately BP (Johns et al., 2011; Johns, 2013; Osborn et al., 2021). The sensory renal afferents, which are chemosensitive and mechanosensitive in nature, are predominantly located in the renal pelvis and function to relay information to the brain to reflexively modulate sympathetic outflow (Johns et al., 2011; Kopp, 2015; Osborn et al., 2021). Activation of the renal afferent pathway triggers either (1) an excitatory renorenal reflex to evoke sympathoexcitation (Barry and Johns, 2015; Kopp, 2015), which is directed not only toward the kidneys but also toward other organs with dense sympathetic innervation, resulting in increased sympathetic outflow and thus a rise in BP (Patel and Knuepfer, 1986; Grisk and Rettig, 2004; Schlaich et al., 2009); or (2) an inhibitory renorenal reflex to evoke renal sympathoinhibition and eventually a reduction in BP (Colindres et al., 1980; Goulding and Johns, 2015; Kopp, 2015).

Hypertension is often linked to overactivity of renal sympathetic and sensory nerves, though the mechanisms remain poorly understood. Renal denervation (RDN) emerged as a potential therapy for resistant hypertension, with second-generation trials addressing the limitations of earlier studies (Esler et al., 2010; Bhatt et al., 2014) and consistently reporting meaningful BP reductions without serious adverse events (Townsend et al., 2017; Kandzari et al., 2018; Böhm et al., 2020). However, RDN remains unsuitable for patients with renal artery abnormalities like stenosis, aneurysm or dysplasia (Roubsanthisuk et al., 2023). Therefore, refining existing RDN procedures or developing an alternative approach to effectively target the renal nerves to evoke a meaningful reduction in BP remains imperative. Recent studies in dogs (Liu et al., 2023) and humans (de Jong et al., 2018) used renal nerve stimulation (RNS) to map BP responses across multiple sites (Liu et al., 2023; de Jong et al., 2018). While most sites evoked pressor responses, some induced depressor effects or had minimal impact on BP. This technique can enhance RDN by identifying optimal ablation sites while preserving depressor regions. Alternatively, targeting vasodepressor sites with neurostimulation could provide sustained BP reductions.

Device-based neurostimulation is gaining notable traction as a treatment for a range of conditions, including hypertension. For instance, baroreflex activation therapy (BAT) using carotid baroreceptor stimulation has been clinically explored for its antihypertensive benefits (Scheffers et al., 2010). However, neuromodulation of the renal nerves, despite their well-appreciated role in BP regulation, remains an untapped area for translational potential. A promising neuromodulation approach using RNS would maintain the structural and functional integrity of the nerves, preferentially engage the inhibitory renorenal reflex, and minimize the activation of the renal sympathetic nerves.

The spontaneously hypertensive rat (SHR), a genetic model of essential hypertension that replicates many of the clinical features of primary hypertension found in man (Trippodo and Frohlich, 1981), is a remarkable animal model that facilitates invasive investigation of autonomic reflexes and provides a scalable landscape for the development of novel therapeutic strategies to treat hypertension. Several research studies focusing on renal physiology in the SHRs have used electrical RNS as a method to confirm proper dissection of the renal nerves (Fink and Brody, 1979; DiBona and Sawin, 1987; Golin et al., 1996). The bulk of those studies relied on the application of supraphysiological stimuli to the peripheral cut end of the nerve while observing changes in ipsilateral renal blood supply. This approach consistently evoked significant reductions in renal blood flow due to the activation of renal efferent nerves (sympathetic). With the afferent stimulation, to our surprise, there currently are no reports on systemic hemodynamic responses to neurostimulation of the intact or afferent renal nerves in the SHRs. However, in normotensive rats, high-charge afferent RNS has been shown to induce a marked increase in BP (Patel and Knuepfer, 1986). Collectively, these findings suggest that ultrahigh-charge injections are most frequently associated with pressor responses, limiting the translatability of this neurostimulation approach as a potential therapeutic intervention for hypertension.

We have recently developed a neurostimulation model for aortic baroreceptor afferents that can achieve, in the SHRs, clinically significant reductions in mean arterial pressure (MAP) of 20–30 mmHg while minimizing energy consumption and adverse effects associated with high charge injections (Salman et al., 2017; Salman et al., 2022; Salman, 2024a). Given that almost all documented RNS studies that report pressor responses to stimulation have used ultrahigh stimulation parameters (Stella et al., 1984; Patel and Knuepfer, 1986; de Jong et al., 2018; Hoogerwaard et al., 2018; Liu et al., 2023), we hypothesized that a neuromodulation paradigm in the low stimulation range might preferentially trigger vasodepressor responses, providing a novel therapeutic alternative for hypertension treatment. Using neurostimulation parameters in the low stimulation range, the present study aimed to provide proof of concept for the potential of neurostimulation of the renal nerves to prompt a clinically relevant reduction in BP in the SHR model of essential hypertension.

## Methods

### Animals

Adult male SHRs weighing 350–400 g (*n* = 8) were purchased from Envigo, United States. All rats were kept in a controlled environment under a 12-hour light/dark cycle and provided with a standard pellet diet and water *ad libitum*. All experiments strictly adhered to the National Institutes of Health Guide for the Care and Use of Laboratory Animals prepared by the National Academy of Sciences and published by the National Institutes of Health. All protocols were reviewed and approved by the Institutional Animal Care and Use Committee at Case Western Reserve University.

### Surgical procedures

Rats were anesthetized with an intraperitoneal (i.p.) injection of 50 mg·kg^-1^ of sodium pentobarbital (Diamondback, Arizona, United States) and anesthesia was maintained using a continuous infusion (Harvard Apparatus Ltd., Massachusetts, United States) of the anesthetic infused into the right femoral vein. Maintenance anesthesia was calculated at a dose of 10 mg·kg^-1^·h^-1^ of pentobarbital and was delivered in saline at an infusion rate of 2 mL·h^-1^. Toe-pinch was regularly performed to assess the adequacy of anesthesia. Core body temperature was maintained using a heating blanket (T/Pump warm water re-circulator, Stryker Medical, Michigan, United States). Using a dissection microscope (Olympus SZ61 dissection microscope, Olympus Life Science, Massachusetts, United States), the right femoral artery was catharized for BP measurement and calculation of MAP. Heart rate (HR) was derived from the pulsatile arterial pressure signal. A ventral cervical incision was performed, and the trachea was intubated using polyethylene tubing to facilitate spontaneous breathing.

A flank skin incision was performed to expose the left kidney and its renal nerve. Approximately 2-mm segment of the left renal nerve was dissected at the point where the nerve coursed between the lower abdominal aorta and the left renal artery. The nerve was then gently placed on custom-made bipolar silver electrodes (interelectrode distance of ≈1 mm) and embedded in a silicone elastomer (Kwik-sil^®^, World Precision Instruments, FL, United States). The bipolar stimulating electrodes were placed such that the anode was distal to the cathode to minimize distal propagation of action potentials and, favorably direct the current toward the brain (Stauss, 2017). The extended wires of the electrodes were then sutured to the flank muscle to stabilize the electrodes. The flank muscle was then sutured to close the dorsal incision and the external skin incision stapled using wound closure clips. The electrodes were then connected to a square pulse stimulator (S88 Dual output square pulse stimulator, Grass Technologies Product Group, United States) using a stimulus isolation unit (Grass Instrument Co. Model PSIU6 Photoelectric Stimulus Isolation Unit, Grass Technologies Product Group, United States). The used stimulation system delivered a monophasic electrical current, and corresponding voltage traces were recorded using a digital oscilloscope (Yokogawa Digital Oscilloscope DL708E, Tokyo, Japan). The rat was then rolled over on its back and an abdominal incision was performed. The bowel was carefully retracted to the right side and a small segment of the lower abdominal aorta distal to the renal arteries was freed of surrounding connective tissue. A perivascular flow probe (TS420 Perivascular Flow Module, Transonic System Inc., New York, United States) was then attached to the aorta to record aortic (hindquarter) blood flow (HQF) and calculate hindquarter resistance (HQR). Through the same ventral abdominal incision, the left kidney was exposed and a needle-type laser doppler flowmeter (Transonic System Inc., New York, United States) was placed into the renal cortex (depth about 1–1.5 mm below the capsule) to record renal cortical blood flow (RCF) and calculate renal cortical vascular resistance (RCR). The bowel was then carefully positioned back in place and covered with a piece of saline-soaked gauze to minimize ongoing fluid loss during the procedure.

All data traces were acquired using CED 1401 data acquisition system (Power3A CED 1401, Cambridge Electronic Design Ltd., Cambridge, United Kingdom). Once the surgical preparation was completed, rats were allowed a ≈ 30-minute stabilization period before undertaking the neurostimulation protocol described below.

### Neurostimulation protocol

Neurostimulation parameters were in accordance with our previous studies (Salman et al., 2017; Salman et al., 2022) but with a slight modification based on prior pilot testing (data not shown). Left RNS was delivered at a constant pulse intensity and width of 0.5 mA and 0.5 ms, respectively. Variable pulse frequencies of low (2.5 Hz) and moderate (5 Hz) stimulation levels were used. All stimulations were performed continuously for 30 s while recording reflex responses in MAP, HR, HQF and RCF. All variables were allowed to return to baseline pre-stimulus levels (2–3 min) before delivering the next stimulus. The order of frequencies was randomized throughout the experiments. The stimulation protocol was conducted once unless adjustments to flow signals were needed due to probe miscontact. At the end of the stimulation protocol, rats were euthanized with an intravenous injection of potassium chloride. All experimental procedures and protocols were completed within ≈3 h.

### Data analysis

Results were expressed as mean ± standard error of mean (SEM). All data were analyzed offline using Spike 2 software (Cambridge Electronic Design Ltd., Cambridge, United Kingdom) and GraphPad Prism (GraphPad Prism software v10 Inc., La Jolla, CA, United States).

Raw data traces were converted into 5-second bins and mean values were plotted against time in seconds as 40-second baseline followed by 90 s from when the stimulus was applied. VR was calculated by dividing MAP values by regional blood flow (BF) measures, as described previously (Possas et al., 2006; Salman et al., 2020; Salman et al., 2022):

Baseline variables were recorded over a 40-second period prior to the undertaking of the neurostimulation protocol. Absolute and percentage changes in MAP, HR, HQF, RCF, HQR and RCR in responses to RNS were measured relative to an immediate 40-second baseline prior to the application of each electrical stimulus. Time to peak was defined as the time in seconds required to achieve the maximum response to RNS.

To assess the level of dependence of MAP responses on concomitant changes in HR and VR, MAP responses across both stimulation frequencies were correlated with pooled bradycardic and HQR responses using Pearson correlation followed by stepwise linear regression.

A two-way ANOVA followed by Bonferroni’s correction was used to identify differences in the response variables to each stimulation frequency relative to baseline levels. A paired t-test was used to compare the time to peak response and the overall changes in hemodynamic responses between the two stimulation frequencies (2.5 and 5 Hz). Significance was defined as *P* ≤ 0.05.

## Results

**FIGURE 1:**
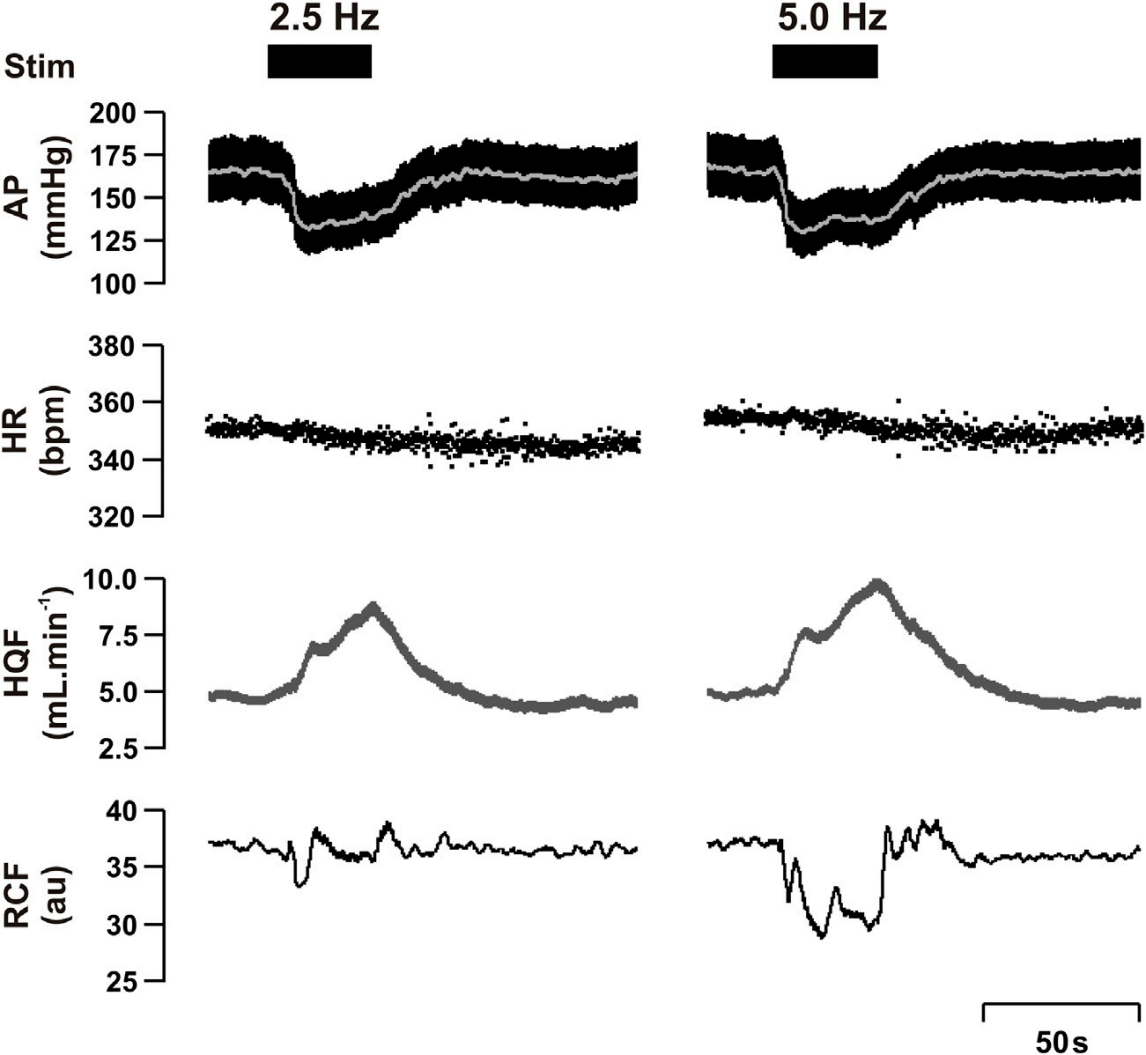
Representative raw data traces showing the effect of 2.5 Hz (Left panel) and 5.0 Hz (right panel) renal nerve stimulation (RNS) at 0.5 mA pulse amplitude, and 0.5 ms pulse width for 30 s in one anesthetized spontaneously hypertensive rat. Stimulation (Stim), arterial pressure (AP), heart rate (HR), hindquarter blood flow (HQF) and renal cortical blood flow (RCF).

Baseline hemodynamic measures are presented in Table 1. A raw data trace of recorded hemodynamic variables is shown in Figure 1.

**TABLE 1:** Baseline hemodynamic measures in anesthetized spontaneously hypertensive rats (SHRs).

Parameter	Baseline
MAP (mmHg)	160 ± 6
HR (bpm)	363 ± 8
HQF (mL·min^-1^)	5.2 ± 0.3
HQR (mmHg·min·mL^-1^)	31 ± 2
RCF (nu)	38 ± 2
RCR (mmHg·nu^−1^)	4.3 ± 0.2

MAP, mean arterial pressure; HR, heart rate; HQF, hindquarter blood flow; HQR, hindquarter vascular resistance; RCF, renal cortical blood flow and RCR, renal cortical vascular resistance. Results are expressed as mean ± SEM (*n* = 8).

### Effect of RNS on MAP

**FIGURE 2:**
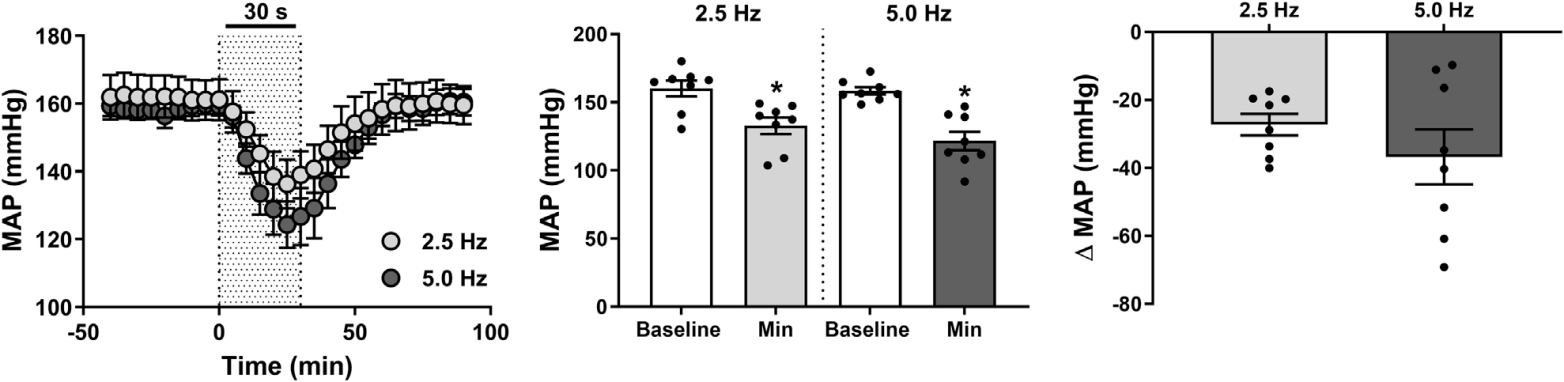
Effect of renal nerve stimulation (RNS) at 2.5 and 5.0 Hz pulse frequency, 0.5 mA pulse amplitude, and 0.5 ms pulse width for 30 s on mean arterial pressure (MAP) in anesthetized spontaneously hypertensive rats (SHRs). The left panel shows the time trend for depressor responses to RNS at 2.5 and 5.0 Hz. The middle panel shows differences in MAP at both frequencies relative to baseline. The right panel shows differences in absolute changes (mmHg) in MAP response to RNS at 2.5 and 5.0 Hz. Results are expressed as mean ± SEM (*n* = 8).^*^ *P* < 0.05 relative to respective baseline.

Both low and moderate frequency stimulation evoked an immediate reduction in MAP relative to baseline (Figure 2), with time to peak MAP reduction being similar at both frequencies (2.5 Hz: 21 ± 3 s vs 5 Hz: 25 ± 2 s; *P* = 0.142). The magnitude of the depressor response was relatively similar at both stimulation frequencies (*P* = 0.157), evoking 27 ± 3 mmHg (17% ± 2%) and 37 ± 8 mmHg (23% ± 5%) drops in MAP at 2.5 and 5 Hz, respectively.

### Effect of RNS on HR

**FIGURE 3:**
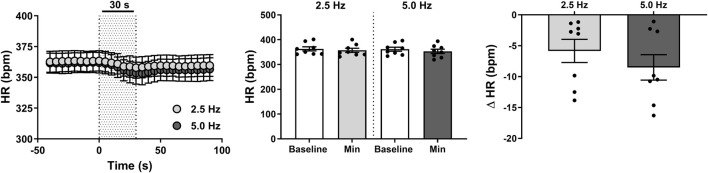
Effect of renal nerve stimulation (RNS) at 2.5 and 5.0 Hz pulse frequency, 0.5 mA pulse amplitude, and 0.5 ms pulse width for 30 s on heart rate (HR) in anesthetized spontaneously hypertensive rats (SHRs). The left panel shows the time trend for bradycardic responses to RNS at 2.5 and 5.0 Hz. The middle panel shows differences in HR at both frequencies relative to baseline. The right panel shows differences in absolute changes (bpm) in HR response to RNS at 2.5 and 5.0 Hz. Results are expressed as mean ± SEM (*n* = 8).

Bradycardic responses (Figure 3) were not markedly different to baseline nor across frequencies (all *P* > 0.05), inducing mere 6 ± 2 bpm (1.6% ± 0.5%) and 9 ± 2 bpm (2.4% ± 0.6%) reductions in HR at 2.5 and 5 Hz, respectively. Likewise, time to peak reductions in HR were similar at both stimulation frequencies (*P* = 0.351), taking place within 27 ± 2 s at 2.5 Hz and 28 ± 1 s at 5.0 Hz.

### Effect of RNS on HQF and HQR

**FIGURE 4:**
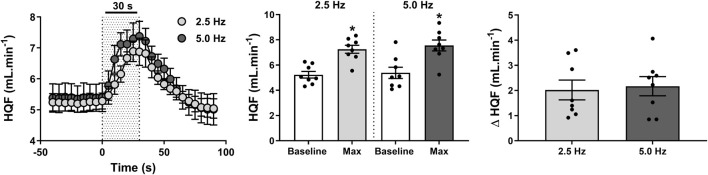
Effect of renal nerve stimulation (RNS) at 2.5 and 5.0 Hz pulse frequency, 0.5 mA pulse amplitude, and 0.5 ms pulse width for 30 s on hindquarter blood flow (HQF) in anesthetized spontaneously hypertensive rats (SHRs). The left panel shows the time trend for HQF responses to RNS at 2.5 and 5.0 Hz. The middle panel shows differences in HQF at both frequencies relative to baseline. The right panel shows differences in absolute changes (mL.min^-1^) in HQF response to RNS at 2.5 and 5.0 Hz. Results are expressed as mean ± SEM (*n* = 8). ^*^ *P* < 0.05 relative to respective baseline.

RNS induced significant increases in HQF (Figure 4) compared with baseline measures (both *P* < 0.01), with peak changes achieved within 22 ± 2 s at 2.5 Hz and 24 ± 2 s at 5.0 Hz. Interestingly, overall increases in HQF remained comparatively similar at both 2.5 Hz (2.0 ± 0.4 mL·min^-1^; 41% ± 9%) and 5.0 Hz (2.2 ± 0.4 mL·min^-1^; 44% ± 10%) stimuli (*P* = 0.669).

**FIGURE 5:**
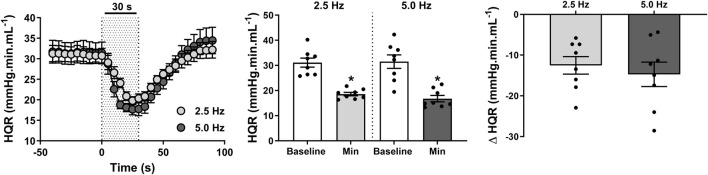
Effect of renal nerve stimulation (RNS) at 2.5 and 5.0 Hz pulse frequency, 0.5 mA pulse amplitude, and 0.5 ms pulse width for 30 s on hindquarter vascular resistance (HQR) in anesthetized spontaneously hypertensive rats (SHRs). The left panel shows the time trend for HQR responses to RNS at 2.5 and 5.0 Hz. The middle panel shows differences in HQR at both frequencies relative to baseline. The right panel shows differences in absolute changes (mmHg.ml.min^-1^) in HQR response to RNS at 2.5 and 5.0 Hz. Results are expressed as mean ± SEM (*n* = 8). ^*^ *P* < 0.05 relative to respective baseline.

Conversely, an instant reduction in HQR (Figure 5) was evoked by both low and moderate frequency stimulations (both *P* < 0.001), with 23 ± 3 s and 24 ± 2 s required to achieve peak changes in HQR at 2.5 and 5.0 Hz, respectively. Most importantly, reductions in HQR in response to 2.5 Hz (13 ± 2 mmHg·min·mL^-1^; 39% ± 5%) and 5.0 Hz (15 ± 3 mmHg·min·mL^-1^; 44% ± 6%) RNS remained comparable (*P* = 0.236).

### Effect of RNS on ipsilateral RCF and RCR

**FIGURE 6:**
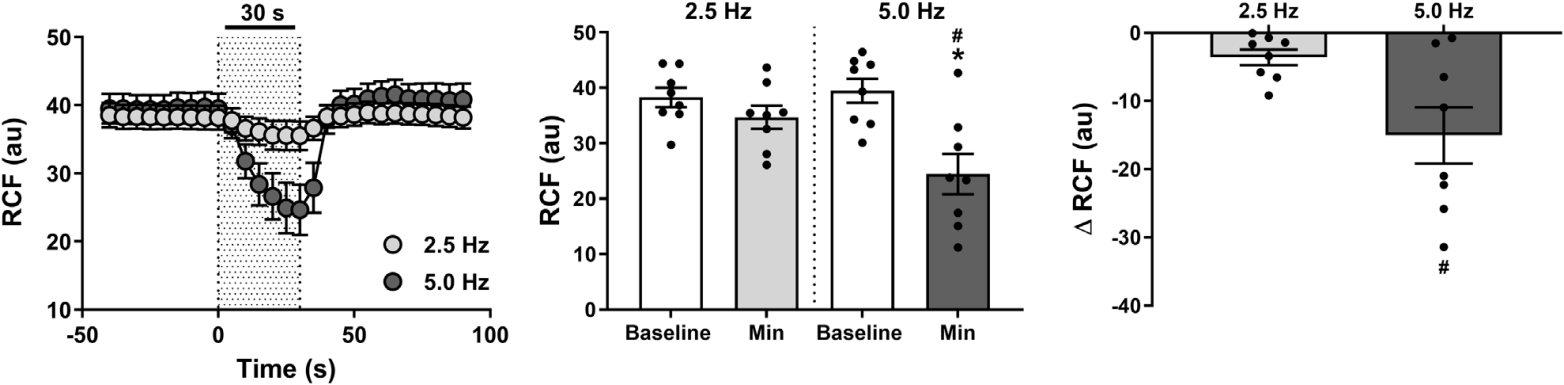
Effect of renal nerve stimulation (RNS) at 2.5 and 5.0 Hz pulse frequency, 0.5 mA pulse amplitude, and 0.5 ms pulse width for 30 s on ipsilateral renal cortical blood flow (RCF) in anesthetized spontaneously hypertensive rats (SHRs). The left panel shows the time trend for RCF responses to RNS at 2.5 and 5.0 Hz. The middle panel shows differences in RCF at both frequencies relative to baseline. The right panel shows differences in absolute changes (nu) in RCF response to RNS at 2.5 and 5.0 Hz. Results are expressed as mean ± SEM (*n* = 8). ^*^ *P* < 0.05 relative to respective baseline and ^#^ *P* < 0.05 relative to 2.5 Hz.

**FIGURE 7:**
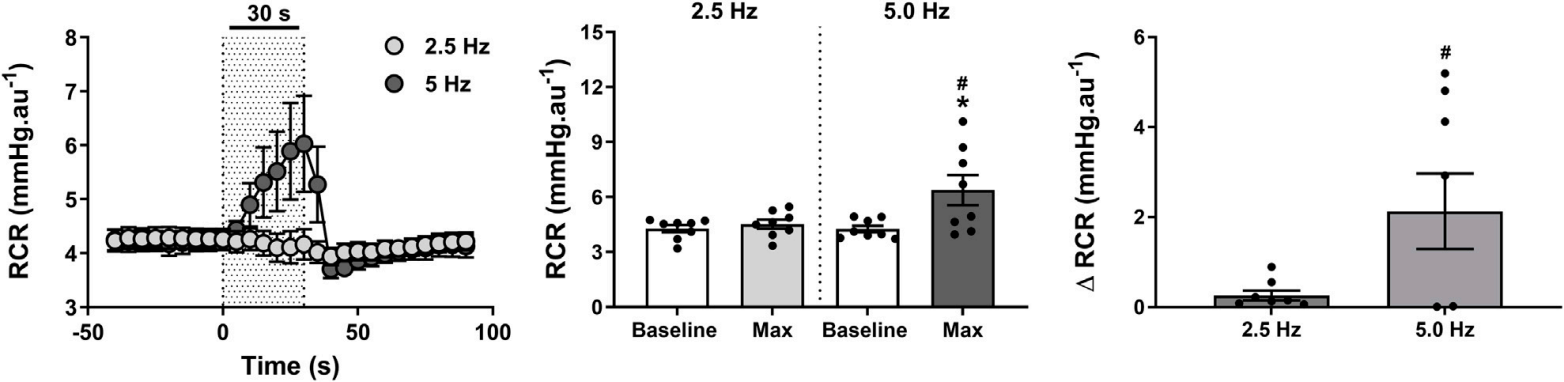
Effect of renal nerve stimulation (RNS) at 2.5 and 5.0 Hz pulse frequency, 0.5 mA pulse amplitude, and 0.5 ms pulse width for 30 s on ipsilateral renal cortical vascular resistance (RCR) in anesthetized spontaneously hypertensive rats (SHRs). The left panel shows the time trend for RCR responses to RNS at 2.5 and 5.0 Hz. The middle panel shows differences in RCF at both frequencies relative to baseline. The right panel shows differences in absolute changes (mmHg.nu^−1^) in RCR response to RNS at 2.5 and 5.0 Hz. Results are expressed as mean ± SEM (*n* = 8). ^*^ *P* < 0.05 relative to respective baseline and ^#^ *P* < 0.05 relative to 2.5 Hz.

An intense reduction in ipsilateral RCF (Figure 6) of 15 ± 4 nu (37% ± 10%) was observed within 19 ± 3 s when the RNS was delivered at 5 Hz (*P* < 0.001). Such reduction became relatively minor (4 ± 1 nu; 9% ± 3%) and indistinguishable from baseline (*P* > 0.999) when the stimulus frequency was lowered to 2.5 Hz (Figure 6). These changes consequently contributed to a marked increase in RCR (Figure 7) of 2.1 ± 0.8 mmHg.nu^−1^ (52% ± 20%) at 5.0 Hz (*P* = 0.009), which was otherwise insignificant (*P* > 0.999) at 2.5 Hz (0.3 ± 0.1 mmHg.nu^−1^; 6% ± 2%).

### Correlation analysis of hemodynamic variables

**FIGURE 8:**
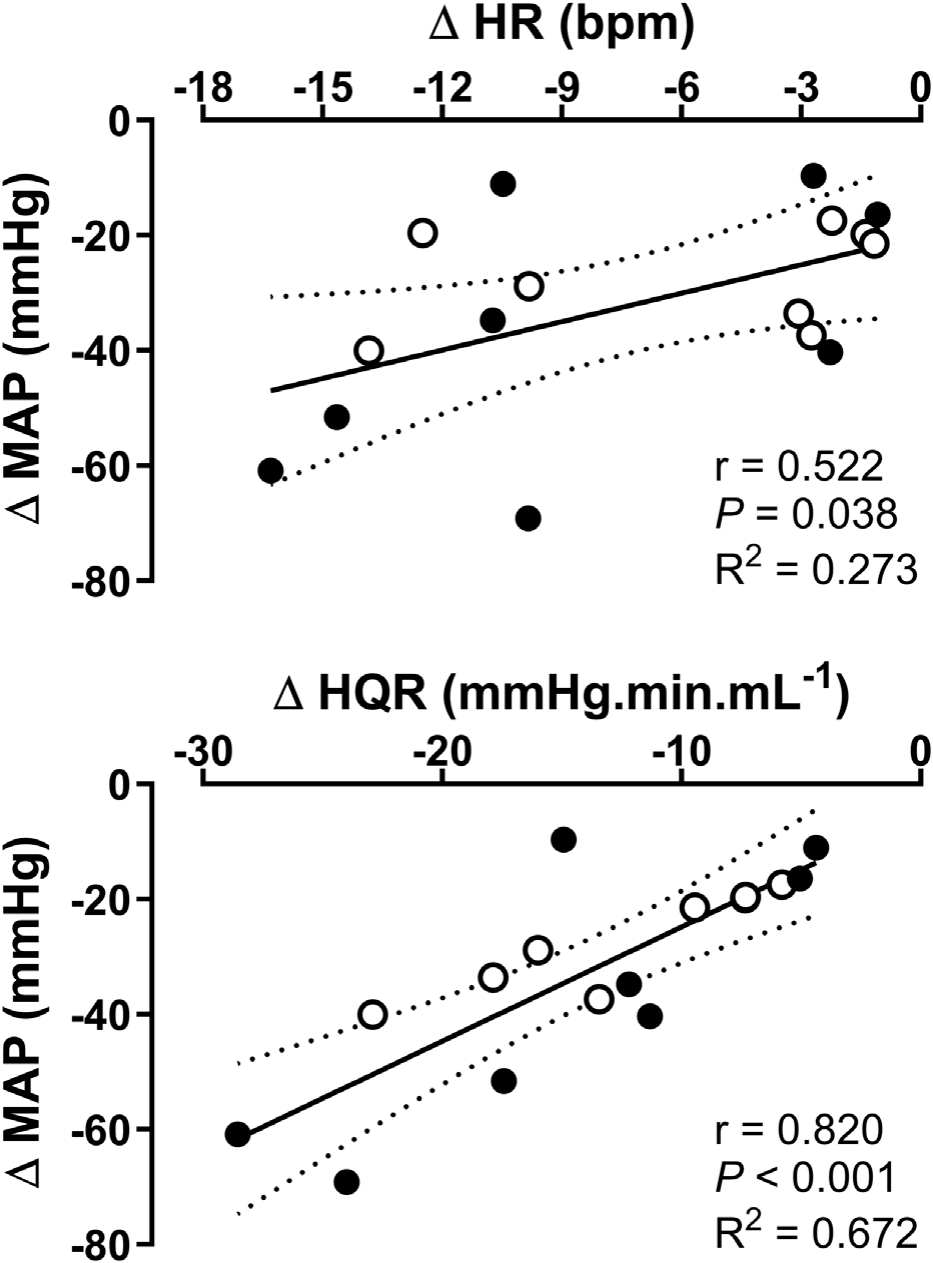
Correlation analysis of the relationship between maximal (upper panel) mean arterial pressure (MAP) versus heart rate (HR), and (lower panel) MAP and hindquarter vascular resistance (HQR) at 2.5 (open circle) and 5.0 Hz (closed circle) stimulation frequency (0.5 mA pulse amplitude, and 0.5 ms pulse width for 30 s) in anesthetized spontaneously hypertensive rats (SHRs, *n* = 8). r, Pearson’s correlation coefficient, and *R* ^2^, regression coefficient.

Correlation analyses indicated a stronger relationship between MAP and VR as opposed to MAP and HR as evidenced by the significant (*P* < 0.001) HQR versus MAP correlation and the higher Pearson’s correlation coefficient (Figure 8).

## Discussion

Stimulation of the renal nerves as a therapeutic modality to treat hypertension may at first glance seem counterintuitive, given the substantial evidence pointing to renal sympathetic hyperactivity as an underlying cause of hypertension (Johns, 2013; Hering et al., 2014) and how renal denervation can evoke significant reductions in BP (Townsend et al., 2017; Kandzari et al., 2018; Böhm et al., 2020). Indeed, the precise mechanism by which RNS works is not well understood, with studies predominantly demonstrating marked increases in BP responses to electrical RNS in both humans and experimental animals. For instance, in conscious normotensive Wistar rats, afferent RNS has been shown to evoke an immediate 10 mmHg increase in arterial BP at 20 Hz, 1.0 ms duration, and 1–3 V (Patel and Knuepfer, 1986). In anesthetized normotensive cats, afferent RNS using 4-ms pulses at a repetition rate of 20–25 Hz and an amplitude of 8–12 V instantly increased MAP by more than 20 mmHg and elevated renal VR (Stella et al., 1984). In anesthetized drug-resistant hypertensive patients admitted for RDN, RNS increased MAP ≥10 mmHg at 20 Hz, 20 mA and 2 ms pulses (de Jong et al., 2018; Hoogerwaard et al., 2018). One major element those studies have in common is the use of supramaximal stimulation parameters to undertake electrical RNS. Such an approach prioritizes the engagement of renal sympathetic nerves directly and/or indirectly via the activation of excitatory renorenal reflex, culminating in BP elevation. While this method offers a quick identification and characterization of the renal nerves, the generated outcomes are far from physiological and have limited translatability into clinical practice, particularly for developing devices aimed at chronically treating hypertension. Accordingly, a key consideration in this study was to use a neurostimulation model with low energy consumption and the lowest charge parameters to elicit an effective reduction in MAP, as we have shown previously (Salman et al., 2017; Salman et al., 2022).

In our studies, RNS using low charge parameters evoked marked reductions in MAP irrespective of the stimulation frequency. It is well known that renal osmo-sensitive and mechanosensitive receptors transduce an afferent signal that triggers the renorenal reflex and reflexively reduces sympathetic outflow (Kopp, 2015). While our stimulation approach bypasses mechanical activation of those receptors it can be postulated that electrical signals arising from our low charge injection protocol may mimic those naturally produced by mechanosensitive activation of those receptors, evoking predominantly a depressor response. Adult SHRs have been shown to lack an inhibitory renorenal reflex in response to mechanoreceptor and chemoreceptor stimulation of the renal afferents (Kopp et al., 1987) and that this deficit could only be restored if hypertension is treated early on (Kopp and Smith, 1989), suggesting that the loss of inhibitory renorenal reflex in the SHRs is related to the maintenance of hypertension. Furthermore, reduced responsiveness of the renal sensory nerves has been encountered in pathological conditions such as hypertension (Kopp, 2015). In our experiments, it is possible that low-level neurostimulation was able to, at least partly, restore the function of the renal afferent fibers and alleviate deficits in the operation of the inhibitory renorenal reflex in the SHRs. Changes in efferent renal sympathetic nerve activity (ERSNA) are thought to directly modulate afferent renal nerve activity (ARNA) independently of hemodynamic factors (Kopp, 2015). For instance, renal pelvic administration of norepinephrine (NE) has been shown to increase ARNA and stimulate the renal pelvic release of substance P (Kopp et al., 2011). This enhanced ARNA, in turn, decreases ERSNA through activation of the inhibitory renorenal reflex, thereby counteracting excessive sympathetic outflow, promoting natriuresis, and reducing BP. Additionally, NE may modulate ARNA at the level of the dorsal root ganglia (DRG), as NE was found to decrease the activity of voltage-gated calcium channels in cultured DRG cells retrogradely labeled from the kidney, through activation of inhibitory α_2_-adrenoceptors (Ditting et al., 2012). This action suppresses neurotransmitter release and dampens signal transmission to central autonomic centers. Thus, the dual role of NE—acting locally at the renal pelvis to enhance ARNA and centrally at the DRG to modulate sensory signal transmission—highlights its complex involvement in the renorenal reflex and overall autonomic and hemodynamic regulation. Further studies are required to elucidate the precise mechanisms by which low-charge RNS induces its antihypertensive effects.

From a physiological perspective, our hemodynamic data offer a mechanistic insight into the primary mechanisms driving the depressor response to RNS. Reflex bradycardic responses were not markedly different relative baseline, suggesting that changes in HR had insignificant contribution to the observed reductions in MAP in response to RNS. However, this finding should be interpreted cautiously due to the well-established depressive effect of pentobarbital on reflex control of HR (Barringer and Buñag, 1990; Watkins and Maixner, 1991). In contrast, vasorelaxation responses, evidenced by the substantial reductions in VR, appear to be the primary driver of the hypotensive response to RNS. Further solidifying this notion is the observation of a stronger correlation between MAP and VR relative to MAP and HR. Such findings are not surprising given the predominant role of VR in BP regulation (Widrow, 2023). Although not directly measured, our VR findings further indicate that reductions in VR are likely contributed to by reflex sympathetic withdrawal in response to RNS given that the vasculature is primarily innervated by the sympathetic nervous system (Bruno et al., 2012; Widrow, 2023). Since renal afferents synapse with interneurons that project to central nervous system sites associated with cardiovascular regulation including the nucleus tractus solitarius (NTS), rostral ventrolateral medulla (RVLM), subfornical organ (SFO), and paraventricular nucleus of the hypothalamus (PVN) (Solano-Flores et al., 1997), it is possible that the applied neurostimulation exerted an inhibitory influence on neurons within the RVLM, a key medullary region responsible for generating changes in efferent sympathetic nerve activity (Kulkarni et al., 2022).

Despite the large body of evidence supporting long-term safety of RDN in the hypertensive population (Townsend et al., 2017; Kandzari et al., 2018; Böhm et al., 2020), the indiscriminate abrogation of renal afferent and efferent neural activities during this procedure may ultimately pose more harm than good, potentially leading to long-term adverse effects or health complications. These concerns are particularly relevant if intact renal innervation supports key physiological functions that remain undiscovered due to our limited understanding of renal innervation, especially regarding the central integration of its sensory input. For instance, recent preliminary data from our laboratory have shown that signals arising from intact renal innervation exert a modulatory influence on respiration in both SHRs (Salman et al., 2021) and Zucker fat rats (Salman et al., 2024), suggesting that the renal nerves may play an essential role in promoting optimal respiratory function. In such circumstances, modulating electrical activity within the renal nerves using neurostimulation, as opposed to RDN, may offer a better therapeutic strategy for hypertension treatment, possibly due to the less invasive nature of procedures involving the implantation of peripheral neural interfaces.

In our experiments, we deliberately stimulated an intact segment of the renal nerve. Such an approach not only minimizes physical damage to the nerve but also maintains both afferent and efferent neuronal activity and consequently preserves innate physiological responses. While activation of the afferent pathway by neurostimulation brought about favorable reflex reductions in BP, an unfavorable concomitant increase in ipsilateral efferent sympathetic activity to the kidney was also noted. This was indirectly inferred by the observed reductions in cortical renal blood flow and the increases in RCR. The latter adverse reaction can render such a neurostimulation approach obsolete as reductions in renal blood supply impair GFR and compromise renal function in the long run (Makanjuola and Lapsley, 2014), limiting the success of this neurostimulation approach in treating hypertension. It is therefore imperative that neurostimulation parameters are carefully adjusted such that a balance between acquiring a meaningful physiological response and avoiding an unexpected or unwanted adverse effect is established. Consequently, a major consideration in this study was to achieve a set of low neurostimulation parameters that can effectively reduce BP whilst mitigating any significant negative impact on ipsilateral renal blood flow. Indeed, the meaningful reductions in MAP in response to a 5-Hz stimulus were associated with marked reductions in ipsilateral RCF. However, when stimulation frequency was lowered down to 2.5 Hz, we were able to induce relatively similar reductions in BP while substantially minimizing the unwanted reductions in ipsilateral renal blood flow, achieving peak RCF responses that were not markedly different from baseline. It has already been established that low-level electrical stimulation of the efferent renal fibers does not markedly alter GFR, renal perfusion pressure, total renal blood flow, or intrarenal blood flow in rats (DiBona and Sawin, 2002) and dogs (Zambraski and DiBona, 1976). However, those effects have never been assessed in intact renal nerves nor have they been reported in the human population.

It might be contended that our study could not definitively determine if the depressor responses to low-level RNS were due to the chosen stimulation parameters or the specific nerve branch used for RNS, which in our laboratory is consistently dissected at the point where the nerve courses between the lower abdominal aorta and the renal artery. Low-level electrical RNS, particularly in intact renal nerves, has been poorly reported. Unless tested on different renal nerve branches, it is challenging to ascertain if the stimulation paradigm alone was the sole driver of the depressor responses in the SHRs. On one hand, the number of studies reporting pressor responses to RNS with high charge injections (Stella et al., 1984; Patel and Knuepfer, 1986; de Jong et al., 2018; Hoogerwaard et al., 2018; Liu et al., 2023) suggests that low-level neurostimulation may indeed be the underlying reason for the observed reductions in MAP. On the other hand, recent evidence has demonstrated that some RNS sites prompted a reduction in BP despite using high charge parameters (de Jong et al., 2018; Zhou et al., 2022; Liu et al., 2023), suggesting that site selection matters when aiming for a depressor response to RNS, possibly due to inherent variation in the exact ratio of afferent to efferent innervation (Liu et al., 2023). Nevertheless, the premise that neurostimulation of the renal nerves can be implemented such that a preferential depressor response is elicited is a remarkable outcome with enormous translational potential for hypertension treatment.

In conclusion, low-level RNS delivered at the juncture where the nerve courses from the lower abdominal aorta to the renal artery can produce clinically meaningful reductions in BP (≈30 mmHg) in the SHR model of hypertension. Physiologically, the observed depressor responses are primarily driven by concurrent reductions in peripheral VR. Adjusting stimulus levels can achieve the desired hypotensive responses without compromising ipsilateral renal blood supply, a common issue with direct renal sympathetic fiber stimulation. Collectively, these findings suggest that low-level RNS may offer a promising therapeutic alternative for hypertension treatment. This study lays a solid foundation for further exploratory and mechanistic investigations into this innovative approach, potentially opening new avenues for effective hypertension management.

## Limitations

One limitation of this study was the recording of hemodynamic responses to RNS under anesthesia. Surgical anesthesia is often criticized for its potential to impair cardiovascular and autonomic reflexes. In contrast, studies conducted in conscious models allow for the recording of cardiovascular variables with greater reliability (Salman, 2024b). However, investigations in anesthetized preparations still provide valuable insights that conscious studies cannot, such as the ability to perform lengthy and invasive procedures in a single setup. This is particularly relevant to the current study, as performing these procedures in conscious animal models would likely result in significant pain and distress during recovery.

Another limitation of this study was the brief duration of hemodynamic response tracking during RNS. To address this, further studies in anesthetized preparations with longer stimulation periods, as well as longitudinal studies in reliable chronic setups, are required. Such studies would help evaluate the adaptation of MAP to sustained stimulation and provide valuable insights into the feasibility and safety of translating these findings into clinical applications. However, based on our current findings, it appears unlikely that the BP reduction would persist after the cessation of electrical stimulation, given the rapid rebound of MAP to prestimulus levels.

## Data Availability

The raw data supporting the conclusions of this article will be made available by the authors, without undue reservation.
